# An all-solid-state NO_3_^-^ ion-selective electrode with gold nanoparticles solid contact layer and molecularly imprinted polymer membrane

**DOI:** 10.1371/journal.pone.0240173

**Published:** 2020-10-15

**Authors:** Lei Zhang, Zhengying Wei, Pengcheng Liu

**Affiliations:** State Key Lab for Manufacturing System Engineering, Xi’an Jiaotong University, Xi’an, China; University of Akron, UNITED STATES

## Abstract

To improve the single-layer all-solid-state ion selective electrode’ defects including poor conductivity of PVC sensitive membrane and interference of water layer between substrate electrode and sensitive membrane, a double-layer all-solid-state ion selective electrode with nanomaterial as the solid contact layer and conductive polymer as the ion sensitive membrane was developed. A gold nanoparticles solid contact layer and a nitrate-doped polypyrrole molecularly imprinted polymer membrane were prepared by electrodeposition. The optimal parameters obtained by electrochemical performance test were 2.5 mmol/L HAuCl_4_ electrolyte for solid contact layer and 1800s electrodeposition time for sensitive membrane. The new electrode exhibited a Nernstian response of -50.4 mV/decade and a low detection limit of 5.25×10^-5^mol/L. Potentiometric water layer test showed no water film formed between the gold nanoparticles solid contact layer and nitrate-doped polypyrrole molecularly imprinted polymer membrane. The contact angle between droplet and the surface of solid contact layer was 112.35° and showed good hydrophobic property. Furthermore, the developed electrode exhibited fast response, excellent potential stability and long lifetime. This electrode is suitable for the detection of nitrate concentration in water and liquid fertilizer.

## Introduction

The extensive mode of industrial and agricultural development has led to serious environmental problems [1] including water pollution caused by heavy metals and soil acidification induced by fertilizer accumulation [2]. The concentration determination of heavy metals and fertilizer elements is very important and various techniques have been utilized for the detection. Nie P, et al. [3] used infrared sensor to detect soil nitrogen content and improved the accuracy with partial least square method. Liu Y [4] analyzed the major and trace elements of anhydrous minerals by inductively coupled plasma mass spectrometry (ICP-MS). Keyvan AV and Jafar M [5] developed a farmer-assistant robot to collect the image of crop leaves and distinguish the extent of nitrogen deficiency by color difference. Other methods for analysis of heavy metals and fertilizer elements include ultraviolet spectrophotometry [6], gas chromatography [7] and liquid chromatography [8]. These spectrometry methods exhibit the drawbacks such as complex pretreatment, expensive instruments and long-term detection and analysis so their applications are restricted to a certain extent.

Ion-selective electrodes (ISEs) based on electrochemical principle are the most frequently used due to its inherent advantages such as portability, low cost, simple operation, low-energy consumption and short-period detection [9]. The traditional liquid-contact ISEs need to be improved because the filling liquid should be replenished regularly and the internal reference electrode Ag/AgCl is oxidized easily [10]. Therefore, the all-solid-state ISEs (ASS-ISEs) have been researched extensively. The coated wire electrodes (CWEs) [11, 12] are the initial form of ASS-ISEs, wherein a thin polymeric film with electroactive materials is coated directly onto a conductor such as platinum wire and graphite rod. However, such an electrode always displays low potential stability on account of the “blocking” interface between the electronically conductive substrate and ion sensitive membrane [13]. The introduction of conductive polymers (CPs, containing polythiophene, polyaniline, polypyrrole and polyparaphenylene) as an internal ion-to-electron layer has improved the potential stability by unblocking the charge transfer at electrode's interfaces [14–17]. Nevertheless, the inevitable water layer and oxidation reaction with dissolved oxygen between the CPs and ion sensitive membrane limit the practical application of electrodes modified with CPs-type membranes.

Recently, carbon-based nanomaterials and synthetic nanomaterials used as the solid contact layer due to their excellent conductivity and hydrophobicity have been proven effective [18]. Zeng X [19], by means of hydrothermal synthesis, developed three-dimensional molybdenum disulfide nanoflower, which was used as the solid contact layer of K^+^ ASS-ISE. Li J [20] utilized the crosslinked three-dimensional porous graphene-mesoporous platinum nanoparticle composite as the solid contact layer of Cd^2+^ ASS-ISE, it showed that no water layer existed. The carbonate ion selective electrodes of multi-walled carbon nanotubes as the inner ion-to-electron transducer exhibited good CO_3_^2-^-selectivity in environmental monitoring, and several anions selective electrodes (including NO_3_^-^, NO_2_^-^ and H_2_PO_4_^-^) were developed [21]. When the electrosynthesis polypyrrole/zeolite composite was employed as solid contact layer in K^+^ ASS-ISE, its electrochemical stability and sensitive performance were improved [22]. Although the carbon-based nanomaterials improved the performance of electrodes, the preparation of carbon-based nanomaterials was complex, dangerous and time-consuming. Most researchers adopted drop-coating process to attach the carbon-based nanomaterials on the surface of substrate electrodes, but the solid contact layer was easy to fall off. These influenced the application of carbon-based nanomaterials in ISEs.

The diameter of gold nanoparticles (AuNPs) is 1~100nm, receiving much attention due to its unique properties such as high electron density and surface chemical activity. Its excellent controllable particle size characteristics have important research and practical application value in the field of potentiometric sensors [23–25]. Bhawan N, et al. fabricated a DNA sensor by stamping of layered rGO and rGO/gold nanoparticles/single stranded DNA (rGO/AuNPs/ssDNA) composites over PET substrates [26]. Liang P, et al. utilized dithiothreitol to fine-tune the surface coverage of oligonucleotide-modified AuNPs for perform cocaine detection in 50% urine [27]. AuNPs formed in-situ on a polymer inclusion membrane (AuNPs-PIM) used as a biocompatible sensing platform for bioreceptor conjugation and the signal-output amplification was implemented on the AuNPs-PIM in a label free biosensor [28]. However, AuNPs were mostly used for in-situ labeling of biomedical sensors and rarely applied in ISEs. The composites composed of AuNPs and carbon-based nanomaterials [29] or AuNPs derivatives were used as the ion-to-electron transducers of ISEs, which increased the complexity of preparation. In order to simplify the preparation process, we utilized AuNPs as the solid contact layer in the NO_3_^-^ ASS-ISE.

A great number of ion-sensitive membranes were plastic films prepared by volatilizing solution in which ionophores and polyvinyl chloride (PVC) were dissolved [30–32]. If the PVC content was less, the ion sensitive membrane was thin and easy to crack. On the contrary, the conductivity of ion sensitive membrane was poor, which affected the ion transmission efficiency. The drop-coating process would also affect the uniformity of ion sensitive membrane. Hence, new materials and technologies are still highly demanded for the development of stable ASS-ISEs.

**Fig 1:**
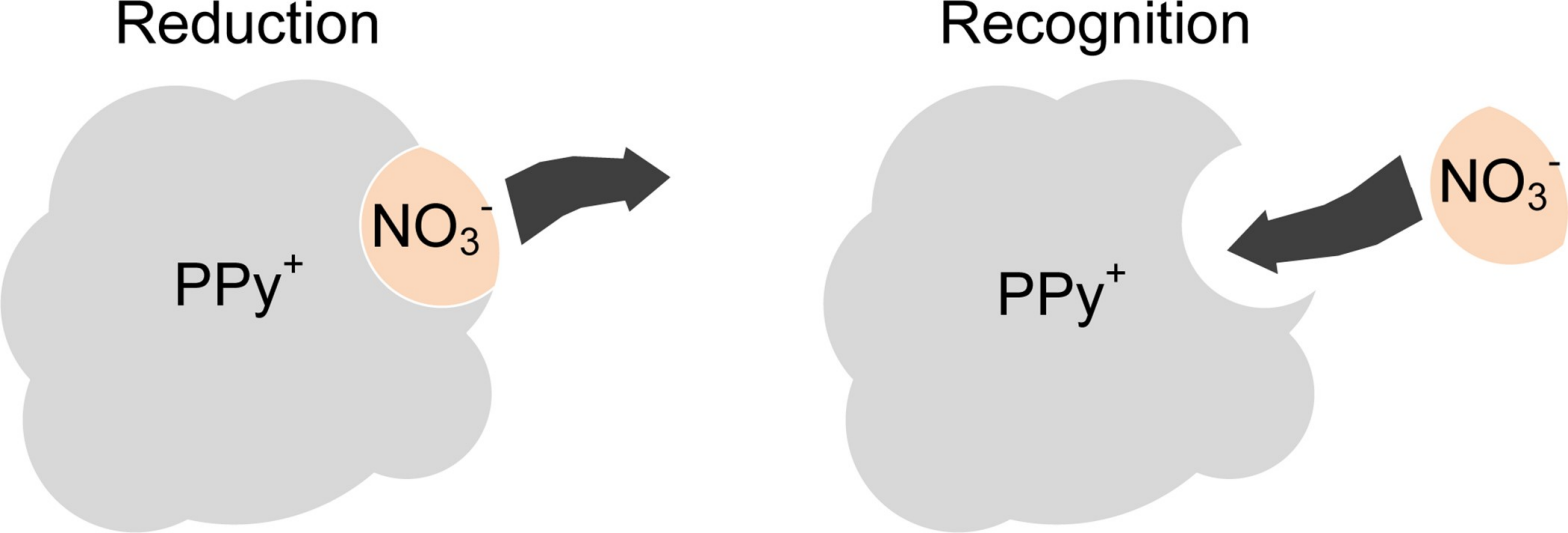
PPy-NO_3_^-^ membrane for selective recognition of NO_3_^-^.

Selective recognition of the target ions based on "molecularly imprinted" technology provides a new method for preparing sensitive membranes [33]. In the process of electrochemical p-doping polymerization of pyrrole monomer, the positive charge in the generated polymer structure must combine with the negative charge to keep electrically neutral. Therefore, NO_3_^-^ with negative charge will be doped into the polypyrrole molecular chain generated by polymerization, forming a corresponding physical space. The specific physical space is "molecularly imprinted key" for the specific recognition of NO_3_^-^. The principle of nitrate recognition in the sensitive membrane formed through nitrate-doped polypyrrole (PPy-NO_3_^-^) is shown in Fig 1. High-performance PPy-NO_3_^-^ membrane can be obtained by electrodeposition. To sum up, the AuNPs solid contact layer and PPy-NO_3_^-^ sensitive membrane provide a low-cost and simple method for the preparation of novel high-performance NO_3_^-^ ASS-ISE.

## Experimental

### Materials and reagents

Glassy carbon electrode (GCE), Ag/AgCl electrode and platinum wire electrode were purchased from Xuzhou Zhenghao Electronics Co., Ltd. Sodium nitrate (NaNO_3_, 99.5% purity), potassium ferricyanide (K_3_[Fe(CN)_6_], 99.5% purity), potassium chloride (KCl, 99.5% purity), pyrrole monomer (99.0% purity), sodium chloride (NaCl, 99.5% purity), potassium phosphate (K_3_PO_4_, 99.0% purity), sodium bromide (NaBr, 99.9% purity), magnesium sulfate anhydrous (MgSO_4_, 99.0% purity) and anhydrous ethanol (99.7% purity) were purchased from Aladdin. Chloroauric acid (HAuCl_4_) solution was of analytical reagent grade from Sinopharm Chemical Reagent Co., Ltd. Deionized water was used throughout.

### Apparatus

Open circuit potential and cyclic voltammetry curve were tested using an electrochemical workstation (PARSTAT 3000, Ametek, U.S.). Solid contact layer and sensitive membrane were electrodeposited by this workstation. Contact angle (CA) between the droplet and the surface of material was measured using an optical contact angle measuring instrument (OCA20, Dataphysics, German). A field-emission scanning electron microscope (FESEM, HITACHI, SU8000) was utilized to characterize the morphology of polymer.

### Electrode preparation

**Fig 2:**
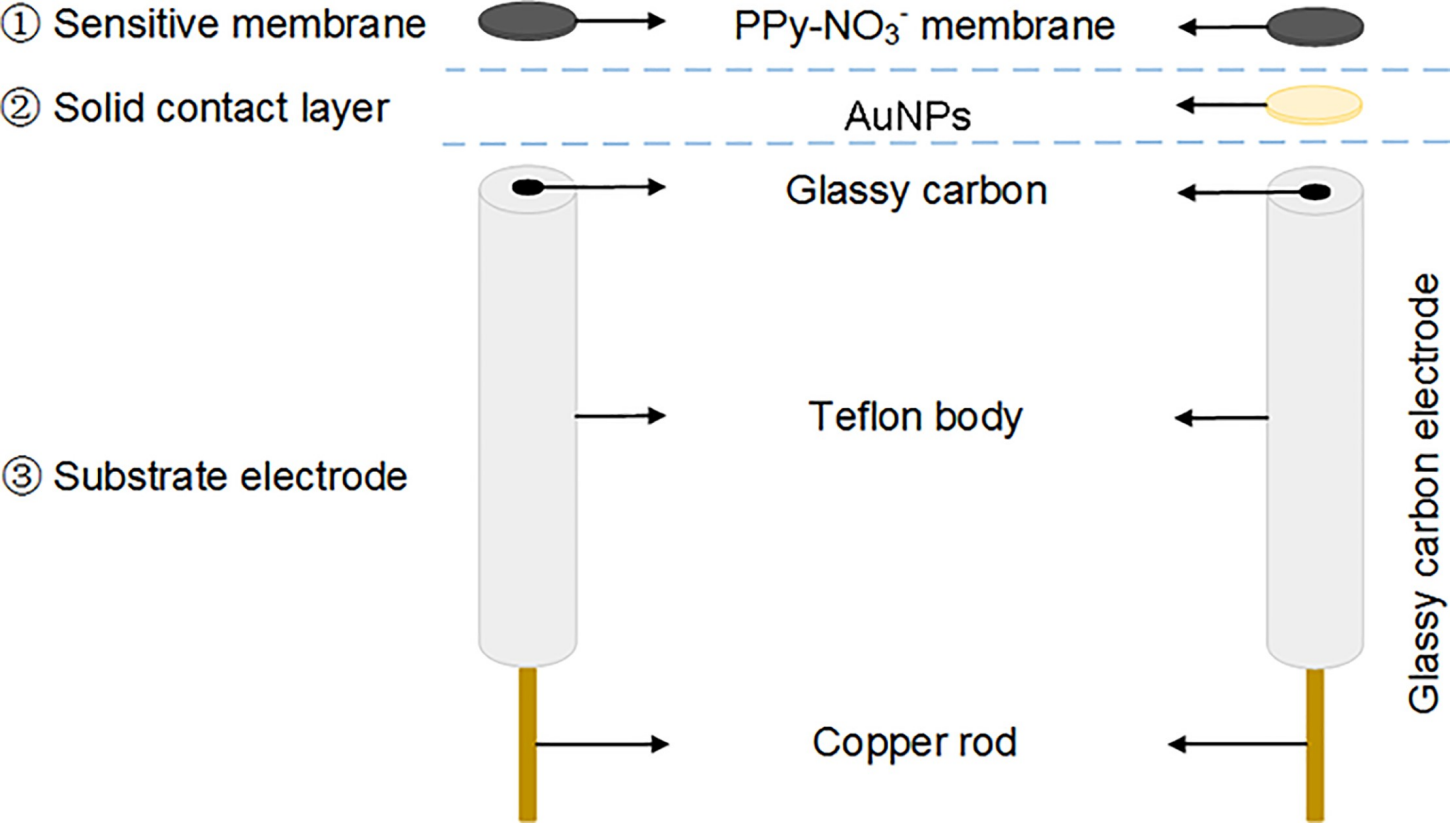
Schematic of NO_3_^-^ ASS-ISE: (left) PPy/GCE and (right) PPy/AuNPs/GCE.

Glassy carbon electrode (GCE, 3mm diameter) was polished with 0.5μm alumina powder and then washed ultrasonically in ethanol (the volume ratio of anhydrous ethanol to deionized water was 1:1) and deionized water. In 1 mmol/L K_3_[Fe(CN)_6_] solution the cycle voltammetry curve (scan potential -0.1V~0.6V, scan rate 50 mV/s) was tested. After a pair of reversible redox peaks appeared and the potential difference between the oxidation and deoxidization peak was less than 80 mV, solid contact layer (AuNPs) was electrodeposited on GCE (AuNPs/GCE) with 2.5 mmol/L HAuCl_4_ by cyclic voltammetry (scan potential -0.9V~0.3V, scan circle 50, scan rate 50 mV/s). AuNPs/GCE was washed and dried. In the mixture of 0.5 mol/L pyrrole monomer and 0.01 mol/L NaNO_3_, PPy-NO_3_^-^ membrane was electrodeposited on AuNPs/GCE (PPy/AuNPs/GCE NO_3_^-^ ASS-ISE) (Fig 2 left) by constant voltage method with 0.7V. For comparison, NO_3_^-^ ASS-ISE without Au NPs layer (denoted as PPy/GCE NO_3_^-^ ASS-ISE) (Fig 2 right) was obtained by covering GCE with the above NO_3_^-^ sensitive membrane. All prepared electrodes were washed and left to dry for 12 hours at room temperature (25±1°C). Then they must be activated in 0.01 mol/L NaNO_3_ solution for at least one day.

## Results and discussion

### Potential response of NO_3_^-^ ASS-ISE

The volume content of sensitive membrane has great influence on the identification efficiency for NO_3_^-^. Different membrane thickness can be formed under different time of electrodeposition. In the three-electrode system with GCE as the working electrode, Ag/AgCl as the reference electrode and platinum wire electrode as the counter electrode, electrodeposition in the mixture of 0.5 mol/L pyrrole monomer and 0.01 mol/L NaNO_3_ was performed for 600s, 1200s, 1800s, 2400s, 3000s and 3600s. After the fabricated PPy/GCE NO_3_^-^ ASS-ISE was cleaned and activated, the potential response was measured in the NO_3_^-^ solution of gradient concentration.

**Fig 3:**
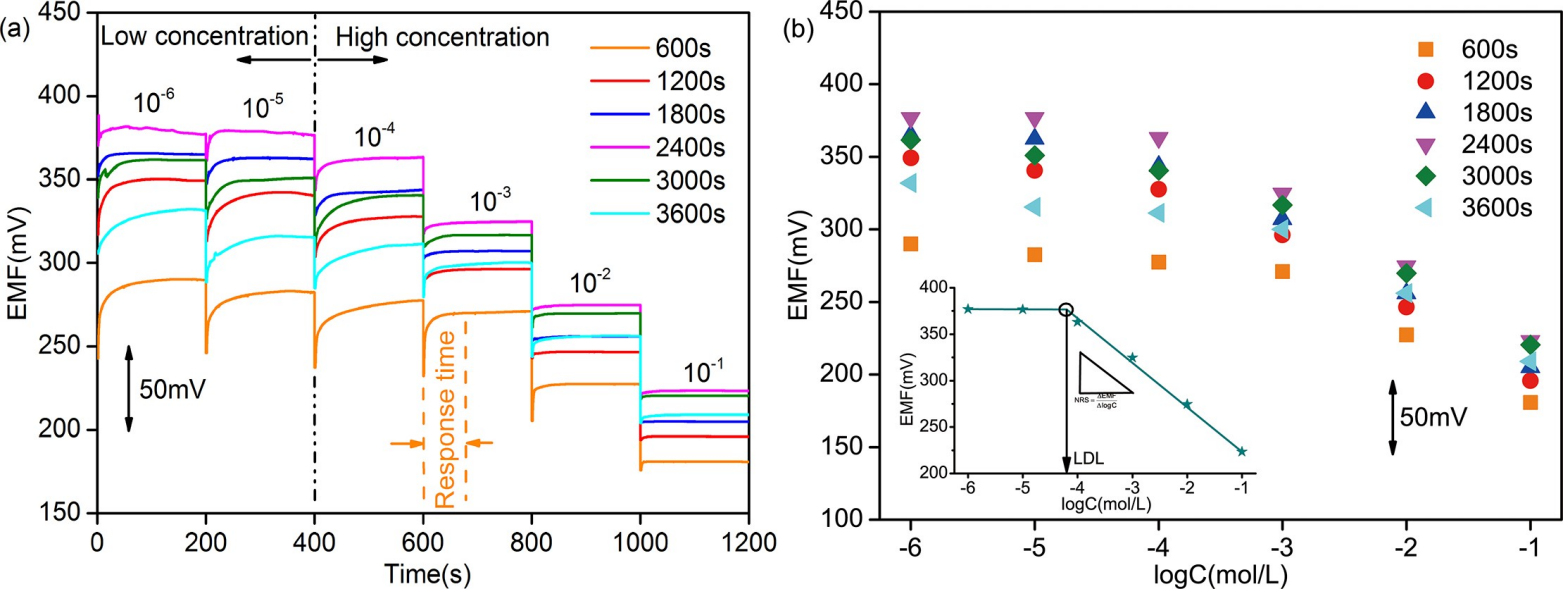
EMF measurement of PPy/GCE NO_3_^-^ ASS-ISE: (a) in NO_3_^-^ solution of increasing concentration and (b) in stable state. All the electrodes were activated in 0.01 mol/L NaNO_3_ solution.

**Fig 4:**
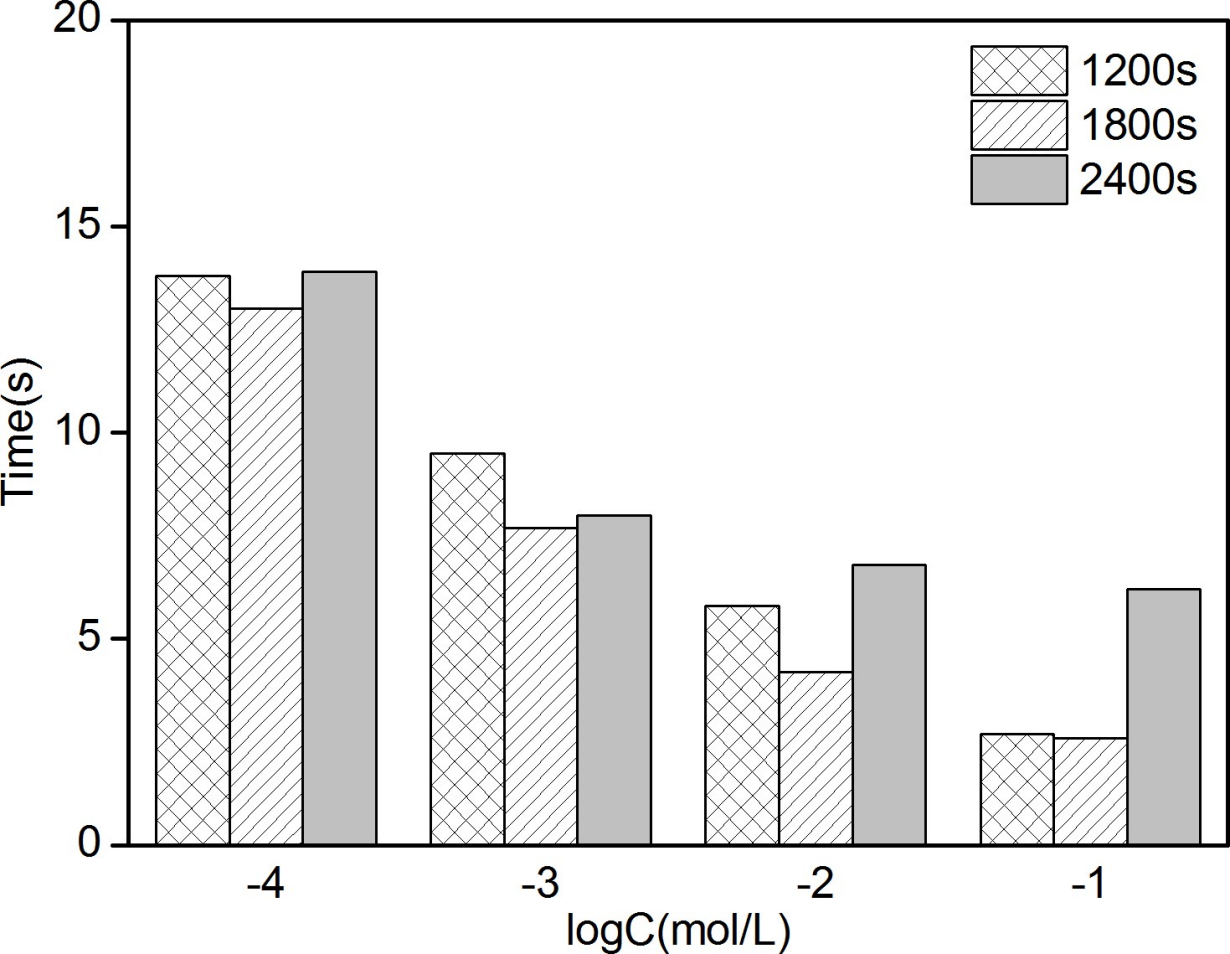
Response times of PPy/GCE NO_3_^-^ ASS-ISE in the linear range of concentrations.

Fig 3(A) shows that the electromotive forces (EMFs) of 6 PPy/GCE NO_3_^-^ ASS-ISEs have significant changes in NO_3_^-^ solutions of different concentrations varying from 10^−6^ to 10^−1^ mol/L, demonstrating these ISEs have good NO_3_^-^-sensitivity. At low concentrations, the EMF of each ISE increases slowly under the condition of concentration change and then reaches its stable value. On the contrary, the EMF quickly reaches a stable value at high concentrations. These can be expressed intuitively in response time. In Fig 3(B), the stable EMF of each ISE in 10^−6^ mol/L solution are basically equal to that in 10^−5^ mol/L solution. At high concentrations, the stable EMF in each concentration shows a decreasing trend with approximate difference, which is in accord with Nernst equation [34]. Hence, the Nernstian response slope (NRS) obtained by data fitting. The lowest concentration in the range of the Nernstian linear response is the lower detection limit (LDL) [35] and the LDL could be calculated from the intersection of the two fitting lines (the inset in Fig 3(B)). The NRSs and LDLs of 6 ISEs are recorded in Table 1. The 3 electrodes electrodeposited with 1200s, 1800s and 2400s have higher detection accuracy and wider working scope because of their larger NRSs and smaller LDLs. Furthermore, the response times of the 3 electrodes in the linear range of concentrations were compared (Fig 4). The comprehensive property of electrode with electrodeposition of 1800s is best, which includes a NRS of -46.7 mV/decade (R^2^ = 0.9912) across a working range of 5.37*10^−5^ ~10^−1^ mol/L and an average response time of 6.875s. The “-” in NRS represents anion.

**Table 1:** The Nernstian response slopes and lower detection limits for 6 PPy/GCE electrodes at the concentration range of 10^−4^~10^−1^(mol/L).

Electrodeposition time(s)	10^−4^~10^−1^(mol/L)	
	NRS (mV/decade)	LDL
		(mol/L)
600	-33.4±1.2	3.55×10^−4^
1200	-44.6±0.9	1.70×10^−4^
1800	-46.7±0.8	5.37×10^−5^
2400	-47.0±1.0	6.31×10^−5^
3000	-40.7±1.5	1.82×10^−4^
3600	-35.1±1.4	1.66×10^−3^

For improving the performance of PPy/GCE NO_3_^-^ ASS-ISE, a solid contact layer was modified between GCE and PPy-NO_3_^-^ sensitive membrane. In the three-electrode system, the gold electrodeposition in the electrolyte of HAuCl_4_ solution with 1 mmol/L, 2 mmol/L, 2.5 mmol/L, 3 mmol/L, 4 mmol/L and 5 mmol/L was performed to form AuNPs/GCE. Then PPy-NO_3_^-^ sensitive membrane was electrodeposited on AuNPs/GCE with 1800s. After the fabricated PPy/AuNPs/GCE NO_3_^-^ ASS-ISE was cleaned and activated, the potential response was measured in the NO_3_^-^ solution of gradient concentration.

**Fig 5:**
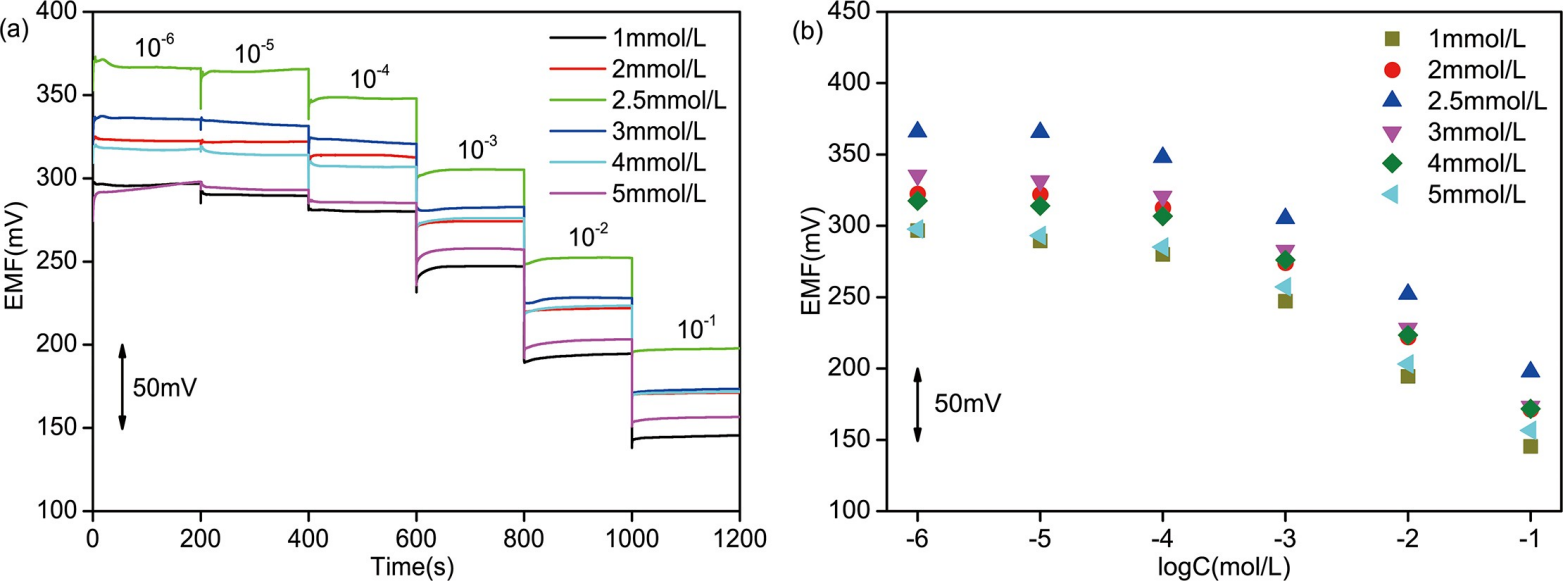
EMF measurement of PPy/AuNPs/GCE NO_3_^-^ ASS-ISE: (a) in NO_3_^-^ solution of increasing concentration and (b) in stable state. All the electrodes were activated in 0.01 mol/L NaNO_3_ solution.

**Fig 6:**
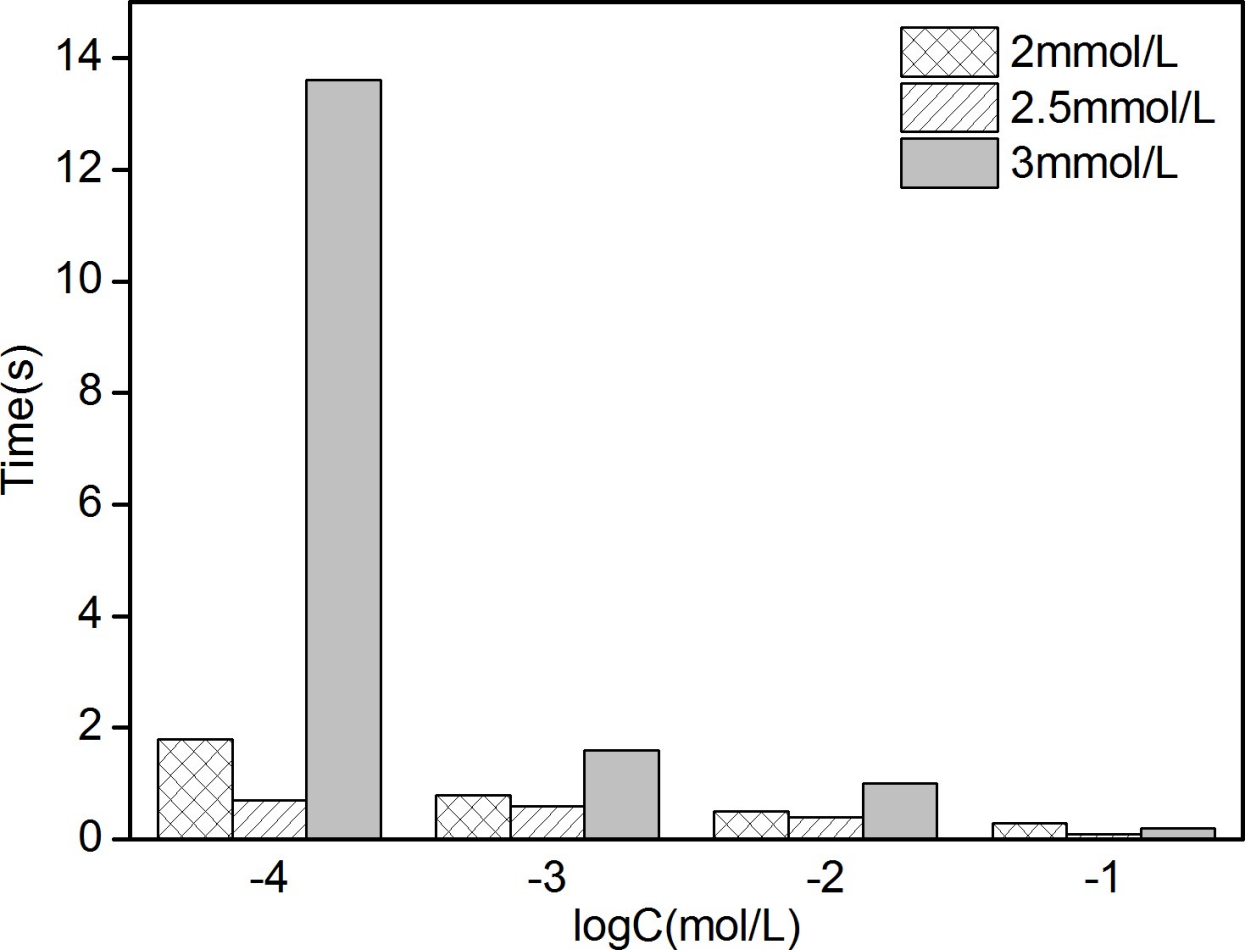
Response times of PPy/AuNPs/GCE NO_3_^-^ ASS-ISE in the linear range of concentrations.

Fig 5(A) shows that the EMF variation trends of 6 PPy/AuNPs/GCE ISEs are the same as that of PPy/GCE ISEs in both low and high concentration solutions. The potential difference of PPy/AuNPs/GCE ISE is larger than that of PPy/GCE ISE at high concentration as shown in Fig 5(B). NRSs and LDLs calculated by the aforementioned method are shown in Table 2. From the three indexes including NRS, LDL and response time (Fig 6), it can be got that the performance of PPy/AuNPs/GCE with gold electrodeposition in 2.5 mmol/L HAuCl_4_ solution is optimal. Compared with PPy/GCE, the average response time decreases by 6.425s and the NRS increases by 7.97%. Hence, the introduction of AuNPs solid contact layer can promote the property of ISE.

**Table 2:** The Nernstian response slopes and lower detection limits for 6 PPy/AuNPs/GCE electrodes at the concentration range of 10^−4^~10^−1^(mol/L).

HAuCl_4_ concentration(mmol/L)	10^−4^~10^−1^(mol/L)	
	NRS(mV/decade)	LDL
		(mol/L)
1	-45.7±1.6	1.20×10^−4^
2	-47.6±0.8	7.76×10^−5^
2.5	-50.9±1.0	5.25×10^−5^
3	-49.6±1.2	9.12×10^−5^
4	-45.8±1.0	1.15×10^−4^
5	-44.0±1.3	1.78×10^−4^

### Selectivity of the NO_3_^-^ ASS-ISE

In order to gauge the response of the ASS-ISE, the selectivity of PPy/GCE and PPy/AuNPs/GCE towards NO_3_^-^ in solutions containing different anions were investigated. The potentiometric selectivity coefficients ($K_{IJ}^{pot}$) of ISEs were determined by the bi-ionic potentials method. The results are summarized in Table 3. It can be seen that the ISEs fabricated in this study have excellent selectivity for NO_3_^-^, and their performance was not affected by the presence of Cl^-^, Br^-^, SO_4_^2-^ or PO_4_^3-^ ions.

**Table 3:** Potentiometric selectivity coefficients of PPy/GCE and PPy/AuNPs/GCE NO_3_^-^ ASS-ISE.

Interfering ion	$K_{IJ}^{pot}$	
	PPy/GCE	PPy/AuNPs/GCE
**Cl**^**-**^	3.1×10^−2^	2.9×10^−2^
**Br**^**-**^	1.4×10^−1^	1.1×10^−1^
**SO**_**4**_^**2-**^	6.1×10^−4^	5.8×10^−4^
**PO**_**4**_^**3-**^	6.4×10^−5^	6.2×10^−5^

### The micrograph of the polymer

**Fig 7:**
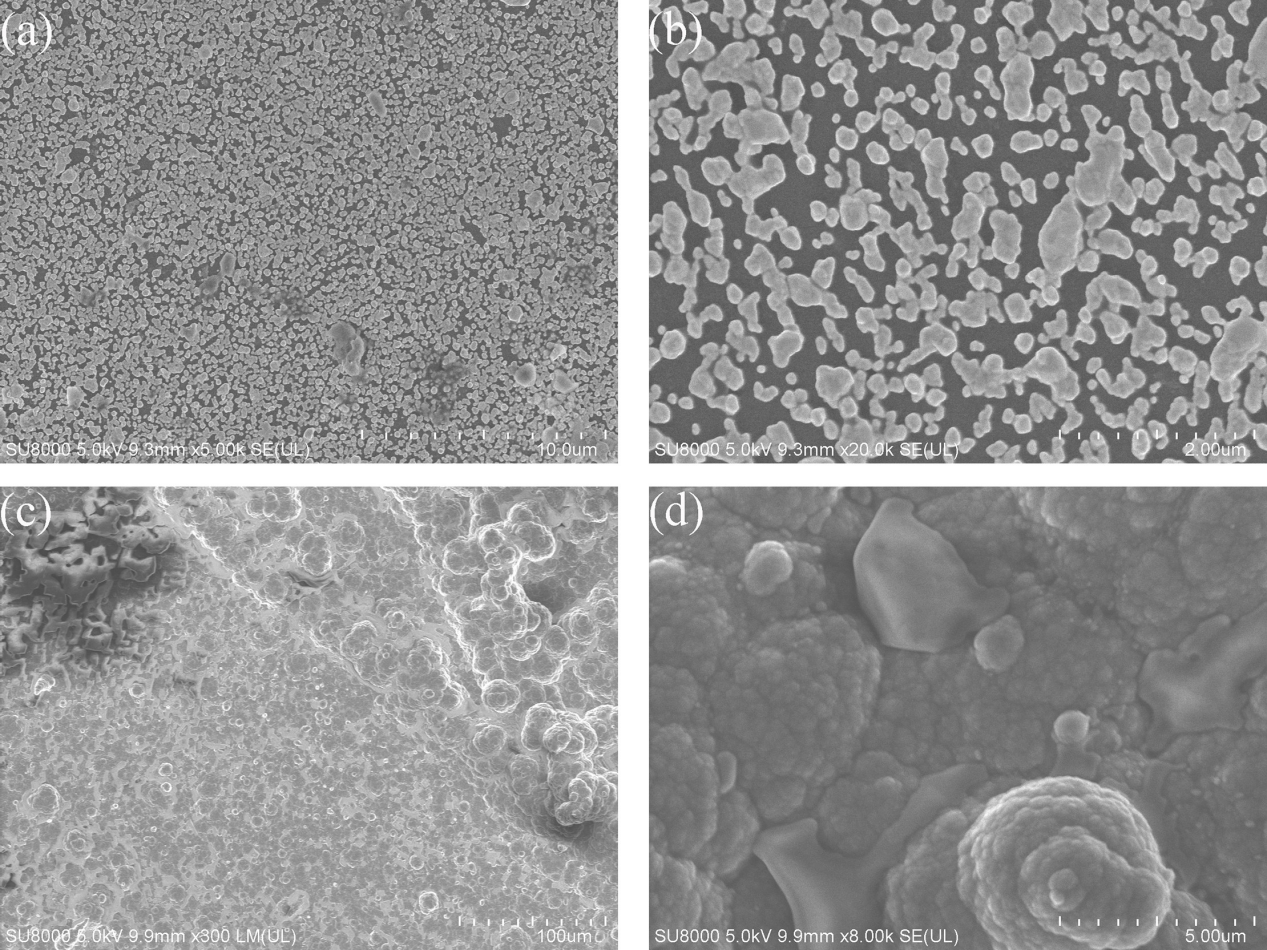
Representative FESEM images of microspheres for (a) AuNPs ×5K (b) AuNPs ×20K (c) PPy ×300 and (d) PPy ×8K.

The morphology of PPy-NO_3_^-^ and AuNPs/PPy-NO_3_^-^ were characterized by FESEM and the typical images are presented in Fig 7. A panoramic picture shown in Fig 7(A) and Fig 7(C) clearly portrays that the morphology of AuNPs and polymer. A higher resolution images are exhibited in Fig 7(B) and Fig 7(D). AuNPs were evenly distributed and PPy looked like cauliflower.

### Water layer test

**Fig 8:**
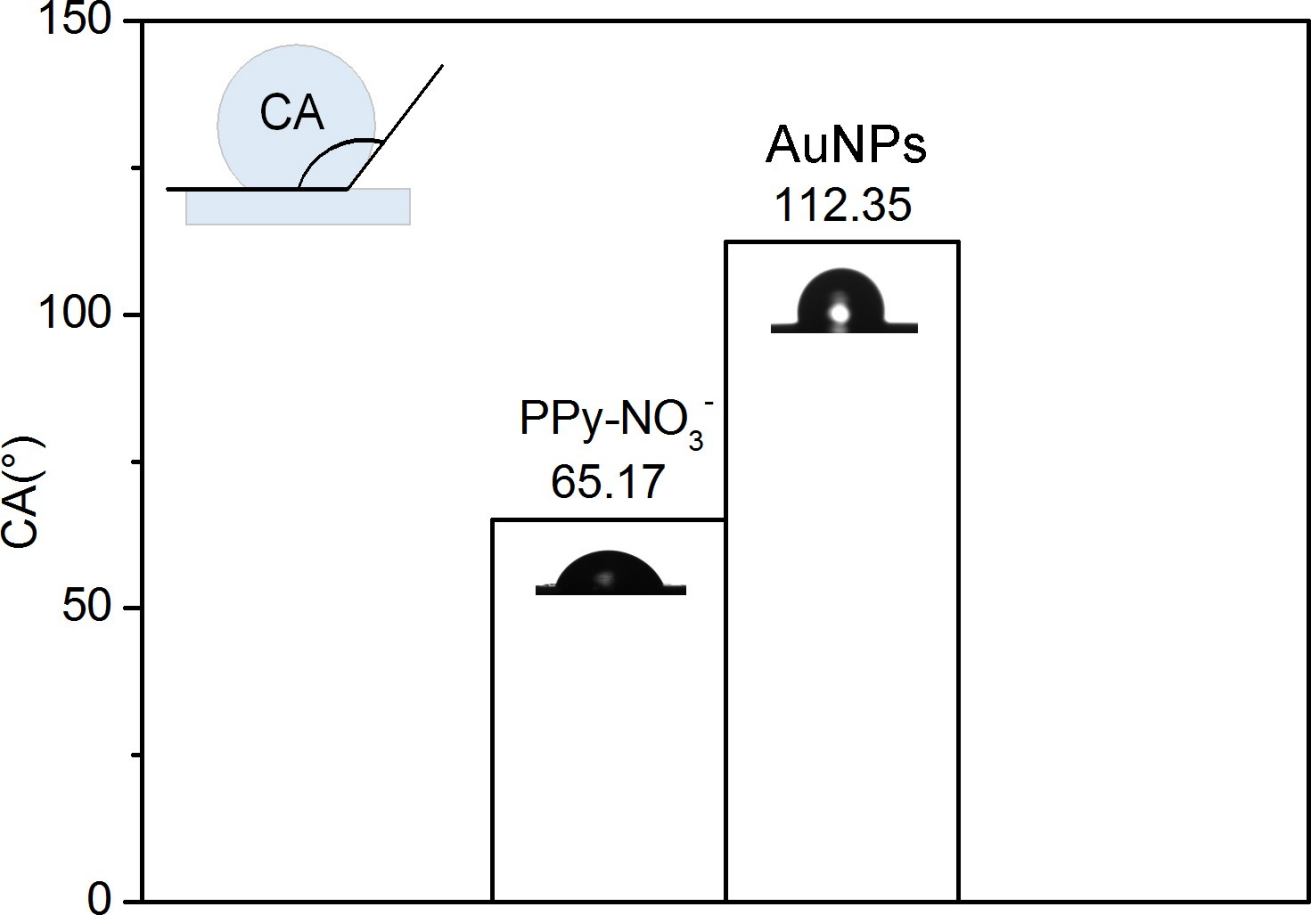
The comparison of contact angles in PPy-NO_3_^-^ membrane and in AuNPs solid contact layer.

Polypyrrole is a hydrophilic polymer [36, 37]. Water molecules can easily pass through the PPy sensitive membrane and form a water layer between the GCE and sensitive membrane, which will affect the performance of the electrode prominently. A droplet was placed on the surface of the PPy sensitive membrane and AuNPs solid contact layer to test the contact angle (CA) (the inset in Fig 8). When the CA is less than 90°, the material has strong hydrophilicity. The hydrophobicity enhances with the CA exceeding 90°. The CA of AuNPs solid contact layer is nearly twice that of PPy selectivity membrane as shown in Fig 8, so as to change hydrophility to hydrophobicity.

**Fig 9:**
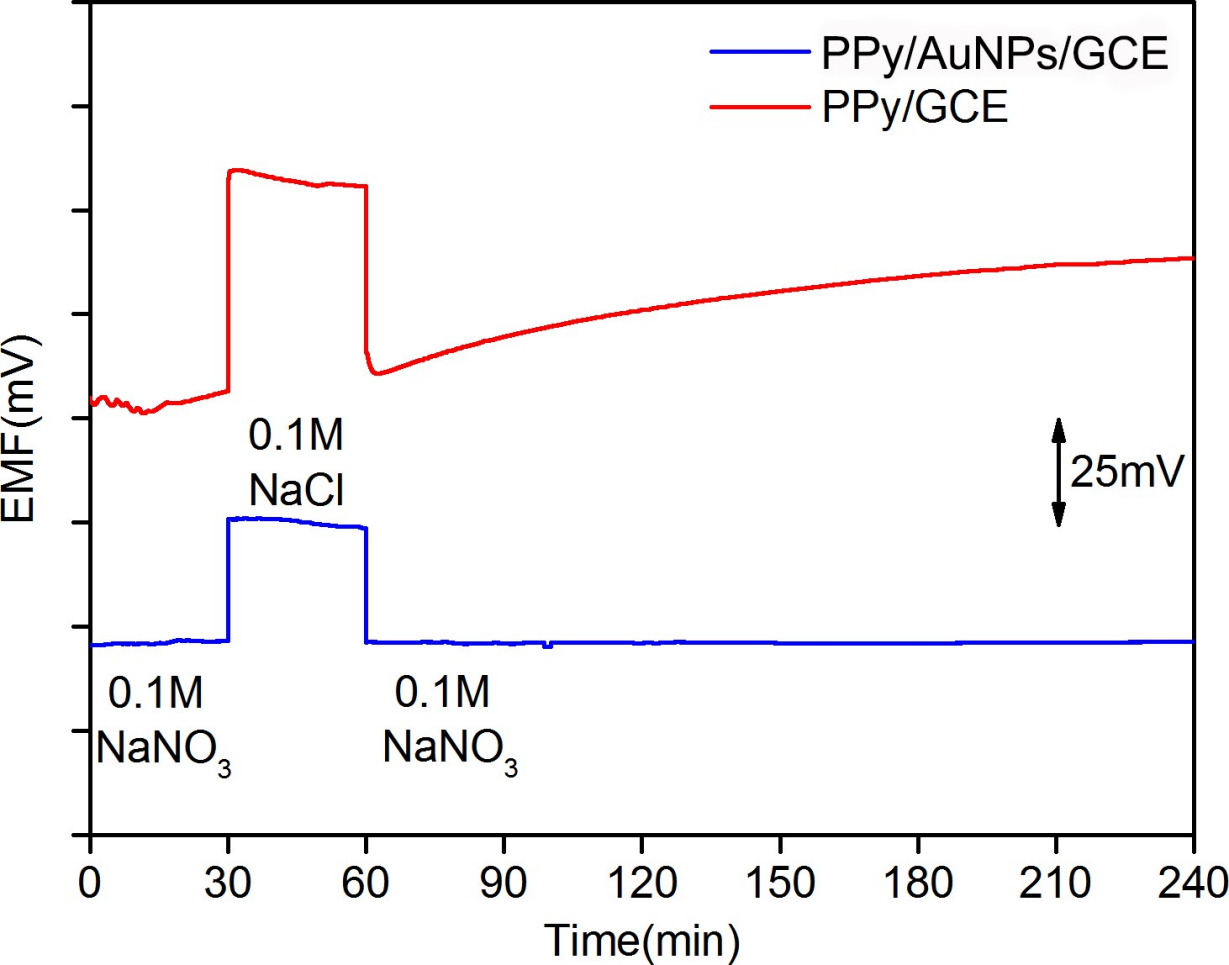
Water layer test of PPy/GCE and PPy/AuNPs/GCE.

As displayed in Fig 9, PPy/GCE and PPy/AuNPs/GCE were immersed in 0.1 mol/L NaNO_3_ solution for half an hour, and the EMFs obtained were stable. When the two electrodes were transferred into 0.1 mol/L NaCl solution, the EMFs of both electrodes changed suddenly. At this moment, the target ion NO_3_^-^ in the PPy sensitive membrane of the two electrodes was rapidly replaced by the interfering ion Cl^-^, which caused the change of the EMF. The potential of PPy/GCE went down obviously. After 30 minutes, the two electrodes were moved back to 0.1 mol/L NaNO_3_ solution. The EMF of PPy/AuNPs/GCE fell back to the initial value rapidly and kept stable. Although the potential of PPy/GCE declined fast, it was not consistent with the initial value and existed a significant drift and responsive hysteresis. These results indicate that there is an interference environment known as the water layer between the sensitive membrane and GCE for PPy/GCE. The velocity of NO_3_^-^ replacing Cl^-^ in the water layer is much smaller than the inverse process. Consequently, the final response potential cannot maintain stable and has serious drift. The changed composition of the water layer with the transport of the corresponding ions from samples which would diffuse through the PPy can affect the stability of potential seriously. However, the AuNPs solid contact layer can effectively inhibit the formation of interference water layer so as to keep stable potential of ISE. These results verify that a reduced water layer is formed between the solid contact layer of AuNPs and the PPy.

### Electrode lifetime

**Fig 10:**
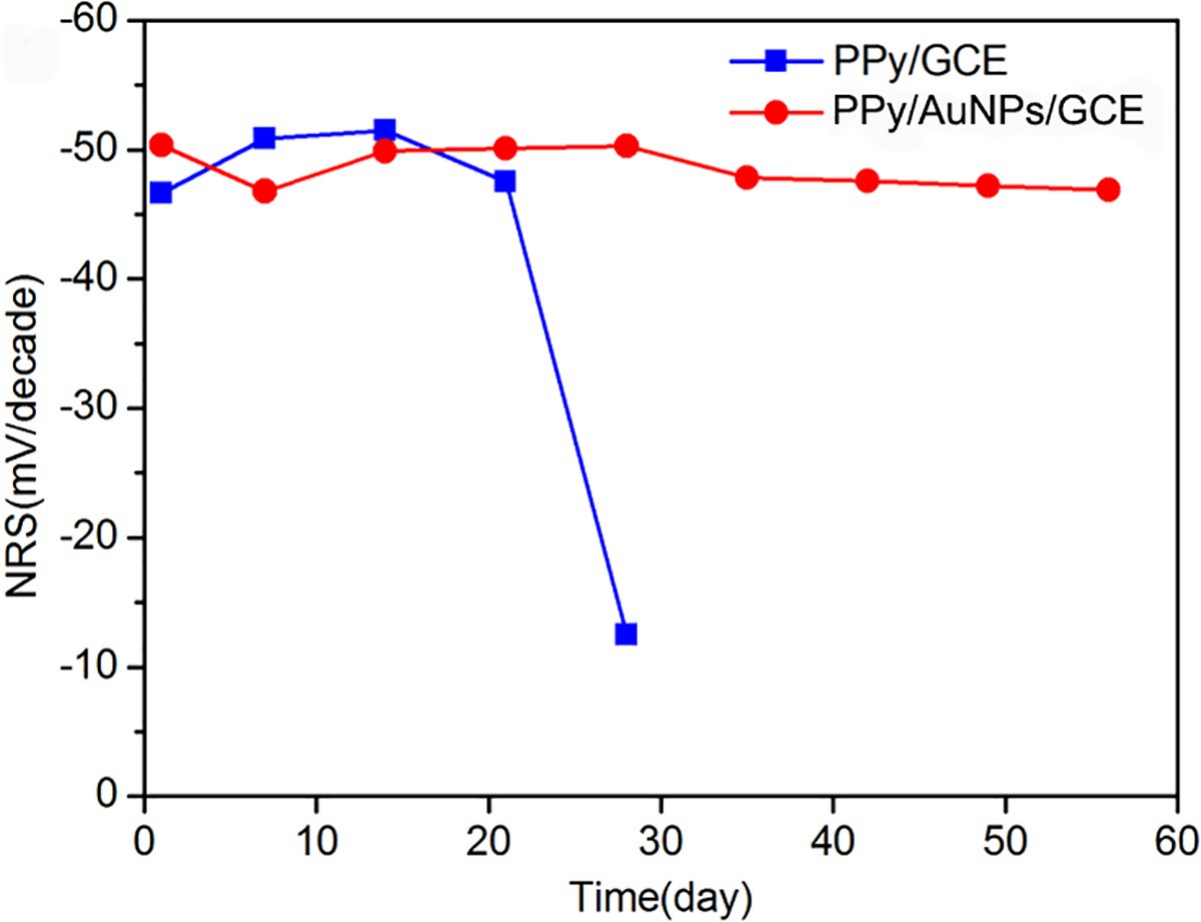
The lifetime test represented by NRS in the range of 10^−4^ to 10^−1^ mol/L.

The EMFs of PPy/GCE and PPy/AuNPs/GCE were measured weekly in the gradient concentration NaNO_3_^-^ solution varying from 10^−4^ to 10^−1^ mol/L, then the NRSs were gotten by fitting calculation. As shown in Fig 10, in the range of 10^−4^ to 10^−1^ mol/L, the NRS of PPy/GCE was degraded extremely after 3 weeks. But the NRS of PPy/AuNPs/GCE was stable for 2 months. Therefore, the AuNPs solid contact layer can prolong the lifetime of NO_3_^-^ ASS-ISE.

### Comprehensive property and follow-up studies

The properties of the two electrodes (No.4 PPy/GCE and No.5 PPy/AuNPs/GCE) manufactured in this paper are compared with those in the literatures. As shown in Table 4, four indexes including NRS, LDL, response time and lifetime represent the performances of electrodes. No.6 has three better indexes, however its lifetime is so short that it cannot be applied in actual detection. Hence, the performances of No. 1 and No. 5 are superior. The lifetime of No.5 lasting for two months is twice that of No. 1, so PPy/AuNPs/GCE is more suitable for continuous detection of nitrogen concentration in nutrient solution.

**Table 4:** Comparison of comprehensive performance for different electrodes.

No.	Linear Range	NRS	LDL	Response Time	Lifetime	Note
	(mol/L)	(mV/decade)	(mol/L)	(s)	(day)	
1	10^−4^~10^−1^	-54.0	2.00×10^−5^	a few	30	[38]
2	10^−4^~10^−1^	-52.0	2.15×10^−5^	—	a few	[39]
3	10^−4^~10^−1^	-52.0	2.50×10^−5^	—	—	[40]
4	10^−4^~10^−1^	-46.7	5.37×10^−5^	6.875	21	PPy/GCE
5	10^−4^~10^−1^	-50.9	5.25×10^−5^	0.45	56	PPy/AuNPs/GCE
6	10^−4^~10^−1^	-56	1.00×10^−6^	62	0.83	[41]

In addition, the NRS and LDL of PPy/AuNPs/GCE can be improved further, and the AuNPs solid contact layer should be optimized to shield the water layer.

## Conclusions

In this work, an advanced and effective sensor based on AuNPs solid contact layer and molecularly imprinted polymer membrane was proposed and further used to fabricate the remarkably sensitive, selective and stable NO_3_^-^ ASS-ISE. Compared with the CWEs, the material preparation and technological process of ASS-ISE were simple. The performance of PPy/AuNPs/GCE was improved with high hydrophobic behavior in contrast to PPy/GCE, a reduced water layer is formed between the solid contact layer of AuNPs and the PPy. A NRS of -50.4 mV/decade in the linear range from 5.25×10^−5^ to 10^−1^ mol/L was obtained. The lifetime could reach 2 months. This work provides a useful approach for implementing AuNPs as solid contact layer in the ISEs, which could expand the applied scope of AuNPs fabricated sensing devices. The molecularly imprinted polymer membrane (PPy-NO_3_^-^ sensitive membrane) improves the disadvantages of PVC sensitive membrane. The better conductivity and stronger cohesive property can be attained.
